# Upcycling Pomegranate Peel into Bioactive Microparticles to Improve Antimicrobial Potential in Apple Juice During Refrigerated Storage

**DOI:** 10.3390/foods15071179

**Published:** 2026-04-01

**Authors:** Elida Coca, Carolina Fredes, Paz Robert, Paula Jiménez, Maria Elsa Pando, Cristina Vergara, Andrés Bustamante, Jesús Lozano-Sánchez, Paula Garcia

**Affiliations:** 1 Departamento de Ciencia de los Alimentos y Tecnología Química, Facultad de Ciencias Químicas y Farmacéuticas, Universidad de Chile, Santiago 8380000, Chile; elida.coca@urp.edu.pe (E.C.); proberts@uchile.cl (P.R.); 2 Departamento de Nutrición y Dietética, Escuela de Ciencias de la Salud, Facultad de Medicina, Pontificia Universidad Católica de Chile, Santiago 7820436, Chile; cpfredes@uc.cl; 3 Departamento de Nutrición, Facultad de Medicina, Universidad de Chile, Santiago 8380453, Chile; paulajimenez@uchile.cl (P.J.); pandosanmartin@uchile.cl (M.E.P.); anbustama@uchile.cl (A.B.); 4 Instituto de Investigaciones Agropecuarias, INIA La Platina, Santiago 8831314, Chile; cristina.vergara@inia.cl; 5 Department of Food Science and Nutrition, University of Granada, 18100 Granada, Spain; jesusls@ugr.es

**Keywords:** pomegranate peel, microparticles, inulin, sodium alginate, carrageenan, apple juice, microbiological stability

## Abstract

Unpasteurized fruit juices are prone to microbiological spoilage by bacteria, yeasts, and molds, creating a need for natural preservatives to extend shelf life without compromising quality. Pomegranate peel extract (PPE) exhibits antimicrobial activity primarily against pathogenic microorganisms; however, its effect on spoilage microorganisms in fruit juices has not been previously studied. This work aimed to compare the microbiological stability of unpasteurized apple juice when adding non-encapsulated PPE or PPE microparticles produced by spray drying with inulin (PPE-IN), inulin combined with sodium alginate (PPE-(IN+SA)), or inulin combined with carrageenan (PPE-(IN+CR)) as encapsulating agents. All microparticle systems showed high encapsulation efficiency (>90%), with PPE-IN reaching 94.08%. For the stability study, PPE microparticles (0.018 to 0.023 g/mL) or PPE (0.009 g/mL) were added to 70 mL of juice and stored at 4 °C for 9 days. MAB, molds, and yeasts were then quantified. At 3, 6, and 9 days, the J+PPE-(IN+CR) treatment showed the lowest MAB, molds, and yeast counts in relation to the non-encapsulated PPE and the other microparticle system. These results indicate that PPE-(IN+CR) microparticles provide superior microbiological stability of apple juice up to day 9, suggesting that the combination of encapsulating agents (IN+CR) could control the release of PPE into the juice.

## 1. Introduction

Fresh fruit juices are high-water-activity foods that contain sugars, organic acids, vitamins, and other nutrients that support microbial growth [1]. The loss of quality in fruit juices can occur due to enzymatic, chemical, physical, and microbiological factors [2]. The primary deterioration of unpasteurized fruit juices is attributed to microorganisms belonging to the juice microbiota, such as molds and yeasts, which can reduce the quality, nutritional value, and shelf life (approximately 3 days under refrigeration) [3,4].

Bacteria are present in low numbers in fresh fruits and vegetables due to their low pH levels. Nevertheless, lactic acid bacteria and Acetobacter have been described as causal agents of spoilage in unpasteurized fruit juices [5].

The microbiological stability of fresh fruit juices has been controlled by various physical and chemical treatments. However, some of these technologies can impact the nutritional and sensory properties of the juice, which are either expensive or not well perceived by consumers [6]. In this context, there is a growing interest in the search for new food preservation methods, with natural food additives obtained from agro-industrial by-products emerging as a notable area of focus [7].

Pomegranate is a balausta fruit with an inedible fraction that corresponds to 57% of fruit weight, in which pomegranate peel (exocarp) represented 10.5% of the fruit’s fresh weight [8]. The global pomegranate production is estimated to be 8.1 million tons [9]. Therefore, an approximate production of 850,500 tons of pomegranate peel, could be estimated as food loss and waste along the food system. The United States Environmental Protection Agency (EPA) proposes a waste food scale, in which “prevent wasted food”, donation, or upcycling are the most environmentally preferred food recovery actions [10]. In this context, valorization of pomegranate peel can involve the extraction of nutrients and bioactive compounds for food purposes, being an opportunity for food recovery, upcycling, and waste reduction [11].

Previous studies have shown that pomegranate peel (PP) is a significant source of phenolic bioactive compounds, including phenolic acids, flavonoids, and hydrolysable tannins (ellagitannins) [12,13,14,15,16]. Among ellagitannins, punicalagin is the major compound found in PP [16]. The antimicrobial activity (AMA) of different types of PPE has been primarily studied in vitro on pathogenic bacteria [17]. We have previously demonstrated the AMA of PPE against *Staphylococcus* aureus [8]. Additionally, research on the efficacy of PPE as an antimicrobial in food applications has focused on pathogenic bacteria in dairy products, meat, and fish [18,19]. To our knowledge, no studies have been conducted on the antimicrobial activity of PPE against spoilage microorganisms in juices. For these reasons, we investigated the effect of PPE on the microbiological stability of fresh, unpasteurized apple juice.

The application of PPE in food matrices may be less efficient than expected because some formulations exhibit unpleasant flavors (astringency), limited solubility, and instability due to exposure to factors specific to the food, such as pH and enzymes, among others [20]. In this context, the encapsulation of PPE could be a potential tool to protect them and control their release in the food matrix.

Different encapsulating agents have been described for the microencapsulation of PPE by spray drying [13,15,21,22,23,24]. In this research, inulin (IN), sodium alginate (SA), and carrageenan (CR) were selected as encapsulating agents due to their different properties, such as solubility, dispersibility, viscosity, and stability at various pH. To the best of our knowledge, IN, SA, and CR have not yet been reported as encapsulating agents in the spray drying of PPE microencapsulation used individually as well as in combinations of them. The main objective of this research was to compare the microbiological stability of fresh apple juice by adding non-encapsulated PPE or PPE-IN, PPE-(IN+SA), or PPE-(IN+CR) microparticles. In addition, we compared the physical, chemical, and morphological characteristics of the microparticles.

## 2. Material and Methods

### 2.1. Materials

Fresh pomegranate (*Punica granatum* L., var. Wonderful) fruits were obtained from a commercial farm located in Vallenar, Chile. IN (Orafti GR^TM^) was *purchased* from Alfa Group (Santiago, Chile); SA was purchased from Prinal (Santiago, Chile); and CR (Quasagel T80) was donated by Quimatic (Santiago, Chile). Punicalagin standard was purchased from Sigma-Aldrich (St. Louis, MO, USA). Tryptone soy agar, trypticase soy agar, water peptone, and Müller-Hinton agar were purchased from PV Equip (Santiago, Chile).

### 2.2. Pomegranate Peel Recovery

PP from fresh fruits were manually separated in laboratory conditions and then were dried by convection in an air-drying tunnel (no brand, built with a Tetlak motor) with a horizontal air flow rate of 2 m/s and 50% recirculation at 60 °C for 16 h. The dried product was ground in a knife mill (Wiley Mill, Model 2, A.H. Thomas Co., Swedesboro, NJ, USA) and then passed through a 20-mesh (840 microns) sieve. The resulting pomegranate peel powder was stored in the dark and kept at room temperature until extraction.

### 2.3. Preparation of PPE

The PPE was obtained by conventional solid-liquid extraction. Briefly, PP (2 g) was macerated in a solution of ethanol and water (38:62 *v*/*v*, 20 mL). The mixture was kept for 3.2 h at 159 rpm in an orbital shaker (JSOS-500 JSR, Seoul, Republic of Korea) and then centrifuged at 4000 rpm for 15 min (Allegra 21R, Beckman, Brea, CA, USA). The supernatant was vacuum filtered using a Büchner funnel. The resulting extract was stored at −20 °C in Falcon tubes. PPE had the following characteristics: moisture content (93.6 ± 0.2%), total solids (6.4 ± 0.1%), total phenolic content (124.3 ± 2.9 mg gallic acid equivalent per gram of dried weight), and punicalagin content (94.2 ± 2.5 mg per gram of dried weight).

### 2.4. Preparation of PPE Microparticles

#### 2.4.1. PPE-IN Microparticles

The microparticles of the PPE-IN system were prepared considering 100 g of solution. IN (1.7–10.8 g) was dissolved in distilled water (84.2–93.3 g) at 70 °C with constant agitation of 200 rpm between 10 to 20 min and then cooled to room temperature (25 °C). 5 g of PPE was added to each preparation and homogenized using a Polytron PT2100 (Kinematica AG, Malters, Switzerland). The solutions obtained were fed into a Mini Spray-Dryer B-290 (Büchi, Flawil, Switzerland) with parallel feed and drying airflow. The spray-dryer was operated at an inlet air temperature between 120 °C and 190 °C, an air flow of 600 L/h, a feed rate of 2.5 mL/min, and a pressure of 5 bar (20 psi). The resulting microparticles were packed in vacuum plastic bags and stored at −20 °C, avoiding contact with light until further analysis was performed.

The experiment was performed using an orthogonal central composite experimental design with 12 runs (4 factorial points, four axial points, and four central points). Independent variables were the PPE/IN ratio (1:0.5–1:2 *w*/*w*) (*x*_1_) and the inlet air temperature (120–190 °C) (*x*_2_). The dependent variable was the encapsulation efficiency (EE) of punicalagin. A response surface methodology (RSM) was applied to determine the optimal conditions for PPE microencapsulation. The data were fitted to a second-order regression model according to the following equation:

y_n_ = bn_0_ + bn_1_x_1_ + bn_2_x_2_ + bn_3_x_1_^2^ + bn_4_x_1_x_2_ + bn_5_x_2_^2^

where *b_n_* are the regression coefficients for the response variable *y_n_*, *x*_1_ is the PPE/IN ratio, and *x*_2_ is the inlet air temperature.

All experiments were conducted randomly to avoid systematic bias. The independent variables’ linear, quadratic forms, and interaction effects on the response variables were considered at a 95% confidence level. Non-significant forms were removed from the equation (Statgraphics Centurion XV, Version 15.1.02, StatPoint, Inc., Warrenton, VA, USA).

#### 2.4.2. PPE-(IN+SA) and PPE-(IN+CR) Microparticles

PPE microparticles with IN in a mixture with SA or CR were prepared using the optimal conditions (i.e., PPE/IN ratio and dryer inlet air temperature) obtained for the PPE-IN system. Note that the same amount of solids was maintained in each studied system. Feed solutions were prepared considering 100 g of solution. IN (9.5 g) was dissolved in distilled water (84.5 g) at 70 °C, maintaining constant stirring. SA (1 g) and the CR (1 g) were dissolved in distilled water at 30 °C and 65 °C, respectively, with continuous stirring (200 rpm) and added to the IN solutions. When the encapsulating agent solutions reached room temperature, PPE (5 g) was added. The solutions were fed into a Mini Spray-Drying equipment (B-290, Büchi, Switzerland). The spray dryer was operated at an inlet air temperature of 166 °C, an air flow rate of 600 L/h, a feed rate of 2.5 mL/min, and a pressure of 5 bar (73 psi).

### 2.5. Characterization of PPE Microparticles

#### 2.5.1. Water Activity (aw)

It was determined using HygroPalm equipment (Rotronic, Hauppauge, NY, USA).

#### 2.5.2. Moisture Content

It was determined using a MA 50.R moisture analyzer (Radwag, Radom, Poland).

#### 2.5.3. Total and Surface Punicalagin Content

Total punicalagin content: A sample (100 mg) of microparticles from each system was mixed with 1.6 mL of High-Performance Liquid Chromatography (HPLC)-grade water, vortexed (1 min), and sonicated at 30 °C for 10 min (ELMA P 60H， Singen, Germany). Then 0.4 mL of methanol was added, vortexed (1 min), sonicated at 30 °C (10 min), and centrifuged at 4000 rpm (6 min) (Allegra 21R, Beckman, Brea, CA, USA). Finally, the supernatant was removed, transferred to a volumetric flask (5 mL), and injected into the HPLC. Assays were performed in triplicate and expressed as mg total punicalagin/g powder.

Surface punicalagin content: A sample (200 mg) of microparticles of each system was treated with 1 mL of methanol, shaken manually (30 s), and then in a vortex (30 s); they were then centrifuged at 1500 rpm (2 min). Finally, the supernatant was filtered (Millipore 0.22 µm) and injected into HPLC. Assays were performed in triplicate and expressed as mg surface punicalagin/g powder.

#### 2.5.4. Punicalagin Content by HPLC

Punicalagin was detected and quantified by HPLC using a Merck Hitachi L-6200 pump, a Waters 996 photodiode-array detector (DAD), and a C18 column (5 μm, 4.6 i.d. × 250 mm, Symmetry, Waters, Ireland) employing the method described by Zhang et al. [24] with some modifications. Briefly, to prepare the mobile phase, Solvent A (0.4% aqueous phosphoric acid) and Solvent B (acetonitrile) eluted according to the following multistep gradient: 0 min (5% B); 10 min (15% B); 30 min (25% B); 35 min (5% B). A 20-µL sample was injected, and the flow rate was set at 1.0 mL/min at room temperature. The monitored wavelength was 360 nm for the detection and quantification of total punicalagin (calculated by the sum of the peak areas of punicalagin A and B), according to a calibration curve (12–200 mg punicalagin/L extract, R^2^: 0.9942). The results of punicalagin content were expressed as mg/g powder. All analyses were performed in triplicate.

#### 2.5.5. Encapsulation Efficiency

EE was calculated using the following equation:

$$\mathrm{EE}\;\%\;=\;\frac{\mathrm{total}\;\mathrm{punicalagin}\;\mathrm{content}\;(\mathrm{mg}/g)-surface\;punicalagin\;content\;\left(\mathrm{mg}/g\right)}{\mathrm{total}\;\mathrm{punicalagin}\;\mathrm{content}\;(\mathrm{mg}/g)}\;\times \;100$$

#### 2.5.6. Microparticle Morphology

The surface structure of microparticles from each system was analyzed by scanning electron microscopy (SEM). The microparticles were covered with gold/palladium using a PS 10E vacuum evaporator and analyzed by an electron microscope (LEO Electron Microscopy Ltd., Cambridge, UK) operated at 20 kV. Scanned images were collected digitally using EDS 7424 software (Oxford Instruments, Oxford, UK).

#### 2.5.7. Microparticle Size

Particle size was determined by laser light diffraction (LLD) using a particle size analyzer (LV 950-V2 Horiba, Kyoto, Japan). The microparticles were dispersed in propylene glycol, and the results were expressed as volume mean diameter (D4.3).

### 2.6. Study of Microbiological Stability

#### 2.6.1. Preparation of Unpasteurized Apple Juice

The raw material consisted of red apples (Malus domestica, var. Fuji) purchased from local markets in Santiago. The fruit was washed and sanitized by immersion in a sodium hypochlorite solution for 3 min. Fresh juice was obtained by pressing raw apples in a slow cold-pressed juice extractor (Ursus Trotter UT-BRONTE150, Yongkang, China). The resulting fresh apple juice (pH 4.0) was transferred to 100 mL sterile glass bottles, refrigerated at 4 °C, and used within 2 h.

#### 2.6.2. The Initial Count of Juice Microbiota

The plate count method was used to count mesophilic aerobic bacteria (MAB) [25] and molds and yeasts [26] in fresh apple juice. 1 mL aliquots of serial dilutions of juice samples were pipetted onto the agar surface and spread around using a sterile glass rod. MAB counts were performed using tryptone soy agar as the culture medium, and the plates were incubated at 35 °C for 24 h. Molds and yeasts were counted using potato dextrose agar as a culture medium. The plates were incubated at 22 °C for 5 days. Assays were performed in triplicate and with three serial dilutions in duplicate for each determination.

#### 2.6.3. Minimum Inhibitory Concentration (MIC)

The MIC was determined to establish the concentration of non-encapsulated and encapsulated PPE to be added to unpasteurized apple juice. For this, non-encapsulated PPE was added in a concentration range of 2–12 mg PPE/mL of apple juice [27]. An amount of PPE was added to tubes of unpasteurized apple juice (10 mL). Samples for MAB count were incubated at 35 °C for 24 h, and samples for fungal and yeast counts were incubated at 22 °C for 5 days. All samples were analyzed in triplicate. The MIC was determined as the lowest concentration of PPE that resulted in a significant reduction in the count of microorganisms [28]. Significant differences were found with a lower count from a concentration of 9 mg PPE/mL of apple juice after 24 h of incubation for RAM and 5 days for molds and yeasts. This concentration was selected for microbiological analysis [29].

#### 2.6.4. Microbiological Stability of Fresh Apple Juice Using PPE Microparticles and PPE

Five systems were considered: J (juice control); J+PPE; J+(PPE-IN); J+PPE-(IN+SA); and J+PPE-(IN+CR).

PPE microparticles (1.31–1.63 g) or PPE (630 mg) were added to 70 mL samples of fresh apple juice in glass bottles, according to the established MIC. PPE and PPE microparticles added to apple juice contain the same content of punicalagin. Samples of fresh apple juice without the addition of PPE were used as a control. Glass bottles were covered, stored at 4 °C, and analyzed after 0 (T_0_), 3 (T_3_), 6 (T_6_), and 9 (T_9_) days for MAB or fungal and yeast counts. Results were presented as a Logarithm of the Colony-Forming Unit per milliliter of juice (Log CFU/mL) at T_0_, T_3_, T_6_, and T_9_. Analyses were performed in triplicate.

### 2.7. Statistical Analysis

Continuous variables were expressed as mean ± standard deviation (*n* = 3). Differences in each variable were analyzed using a one-way ANOVA test for mean comparison. The Tukey HSD (honest significant differences) multiple-comparison test (*p* ≤ 0.05) was applied when significant differences were found. Analyses were performed using Statgraphics software (Statgraphics Centurion XV, Version 15.1.02, StatPoint, Inc., Warrenton, VA, USA).

## 3. Results and Discussion

### 3.1. Microencapsulation of PPE by Spray-Drying

An RSM experiment was conducted to find the optimal conditions for PPE microencapsulation. The EE of punicalagin (83.3% to 95.2%) varied across experimental runs, depending on the PPE: IN ratio and the inlet air temperature. The complete set of experimental conditions and corresponding EE values is presented in Table 1. The statistical analysis using ANOVA revealed that the linear and quadratic forms of the inlet air temperature, the linear form of the PPE/IN ratio, and the interaction between the PPE/IN ratio and the inlet air temperature were significant (*p* < 0.05) on the EE. The model explained 94% of the variability (adjusted R^2^, corrected for degrees of freedom) in the EE of punicalagin, with a residual value of less than 1.7. Furthermore, the lack of fit (2.49) was not significant (*p* = 0.2399), indicating that the mathematical models fit the experimental data well. The quadratic regression equation that describes the effect of the independent variables on the EE was the following:

EE = 39.7443 + 11.8858x_1_ + 0.475612x_2_ + 0.0509524x_1_x_2_ + 0.00110315x_2_^2^

Optimization showed that the highest EE of punicalagin was obtained when using a PPE/IN ratio of 1:2.1 (i.e., the axial (upper) point of the experimental design and total solids of 15.5 g) and an inlet air temperature of 166 °C (i.e., an intermediate value of the experimental design).

### 3.2. Characterization of PPE Microparticles

PPE-IN, PPE-(IN+SA), and PPE-(IN+CR) microparticles were prepared under the optimized spray-drying conditions previously determined. The general physical and chemical characteristics of the microparticles are presented in Table 2, highlighting how the type of encapsulating agent influences the properties of the powders. The physical characteristics showed that the aw of PPE-IN microparticles was higher (0.18) than PPE-(IN+CR) microparticles (0.14). Although PPE microparticles showed certain differences in aw, all values were low (<0.2). This feature is important for PPE microparticle storage and the addition of juice because a low aw prevents potential microbial food spoilage [30]. PPE-IN and PPE-(IN+CR) microparticles also had higher moisture content (3.46 and 3.63%, respectively) than PPE-(IN+SA) with 2.72%. Nevertheless, the moisture content of the PPE microparticles was within the acceptable range for food powders (3–10%). This low moisture content may also favor the storage of PPE microparticles and their future use in food applications.

Therefore, low water activity and suitable moisture content contribute to the physical and microbiological stability of the powder, avoiding agglomeration and preserving bioactive compounds during storage.

PPE microparticles showed differences in total and surface punicalagin content; PPE-IN microparticles had higher total punicalagin (4.45 mg/g) and lower surface punicalagin (0.26 mg/g) contents than PPE-(IN+SA) (3.59 and 0.35 mg/g, respectively) and PPE-(IN+CR) (3.85 and 0.32, respectively), showing significant differences between these last two systems, concerning to PPE-IN.

EE was high (>90%) in all the PPE microparticle systems, which could be attributed to the high solids content of the feed solution and the intermediate inlet air temperature in the dryer chamber. High encapsulation efficiency ensures that the majority of the PPE is protected within the polymer matrix, preventing degradation and enabling controlled release. The higher EE in PPE-IN microparticles (94.08%) could be attributed to their technological characteristics. Note that IN is safe, low-cost, and dispersible in water, and it exhibits low viscosity in concentrated solutions, which varies with temperature [31]. For this reason, various phenolic compounds have been encapsulated with IN using spray-drying, obtaining a high EE (70 to 99%) and a high yield (>70%) [32,33,34,35,36]. For instance, inulin has been used for encapsulating γ-oryzanol by spray-drying, obtaining an EE of 82.63% [28]. More recently, Ogrodowska et al. [37] compared different wall materials and emulsion types for linseed oil encapsulation; when inulin was used in a microemulsion, the EE reached 93.08%, while nanoemulsions with inulin achieved an EE of 98.55–98.82%. In addition, Quintriqueo-Cid et al. [38] investigated the influence of inulin crystallinity on spray-dried quercetin-inulin microparticles. They reported that increasing the crystallinity index of inulin from 2% to 20% raised the encapsulation efficiency of quercetin from 70.5% to 76.4%, while maintaining recovery values above 90%.

SA and CR have been less used in spray drying. These polymers exhibit different technological properties compared to IN, such as lower solubility and higher viscosity at low solid content [39]. Indeed, SA and CR have been incorporated in concentrations < 2% in the feed solution for spray-drying [40,41,42]. Rifna et al. [43] encapsulated PPE using SA by ionic gelation, reaching an EE of 84%. Martinovic et al. [44] encapsulated phenol-rich extracts of grape pomace by spray-drying using SA or in a mixture with gum arabic or gelatin. SA-gelatin achieved the highest EE 95.90–98.01%, whereas with SA, the EE was lower at 70%. Tea extracts were encapsulated with SA and CR by spray drying showed EE of 77 and 93%, respectively [45].

Particle size measurements were based on the assumption that the powders exhibit a spherical shape [46]. For PPE-IN, the mean particle diameter was 5.87 μm, with 90% of the particles below 10.19 μm. PPE-(IN+SA) had a mean diameter of 11.69 μm, with 90% of particles below 22.46 μm, while PPE-(IN+CR) showed a mean diameter of 13.3 μm and 90% of the particles below 26.92 μm. Jayanudin et al. [47] denoted that the particle size of spray-drying microparticles ranges between 1 and 50 µm. Therefore, the particle sizes is aligned with microparticles described for spray-drying. Nevertheless, the higher particle size observed for PPE-(IN+SA) could be explained by the greater tendency to agglomerate produced by the high hygroscopicity of microparticles using SA [48]. Furthermore, the high viscosity of SA or CR feed solutions produces larger droplets during atomization, resulting in a greater particle size [41]. Considerably larger particle sizes (90 μm) were reported by Rifna et al. [43], who encapsulated PPE using spray drying with maltodextrin as the encapsulating agent.

**Figure 1:**
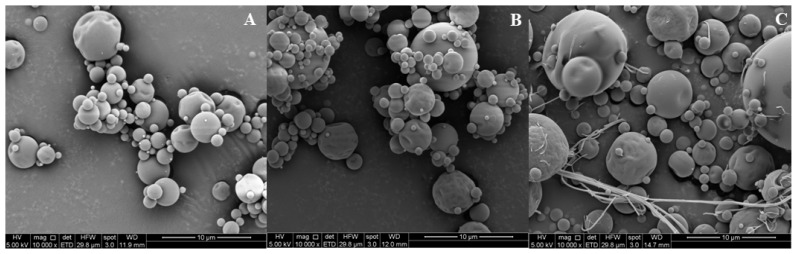
Scanning electron micrographs (×10,000) of pomegranate peel extract (PPE) microparticles obtained under optimal spray-drying conditions: (**A**) PPE-IN: pomegranate peel extract-inulin microparticles; (**B**) PPE-(IN+SA) pomegranate peel extract-inulin-sodium alginate microparticles; (**C**) PPE-(IN+CR) pomegranate peel extract-inulin-carrageenan microparticles. Scale bar: 10 µm.

The morphology of PPE microparticles, visualized by scanning electron microscopy, revealed predominantly spherical shapes with smooth surfaces and the presence of slight surface indentations. A certain degree of agglomeration was observed, consistent with the behavior of spray-dried powders. In the case of PPE-(IN+CR), thin fibrous structures were also detected, likely corresponding to undissolved carrageenan. These morphological features are illustrated in Figure 1.

The observation of similarities in the morphology of PPE microparticles was in agreement with the description by Fang and Bhandari [49], who suggested that the encapsulation method affects both the shape and size of powders. A certain degree of agglomeration observed in SEM photographs can be attributed to collisions between microparticles within the drying chamber (primary agglomeration) and particle collisions that emerge from the drying chamber as recirculating dust (secondary agglomeration) [50]. Nevertheless, a certain degree of agglomeration was in harmony with a previous study on microencapsulation using IN, SA, and CR as encapsulating agents [48].

### 3.3. Microbiological Stability of Unpasteurized Apple Juice

**Figure 2:**
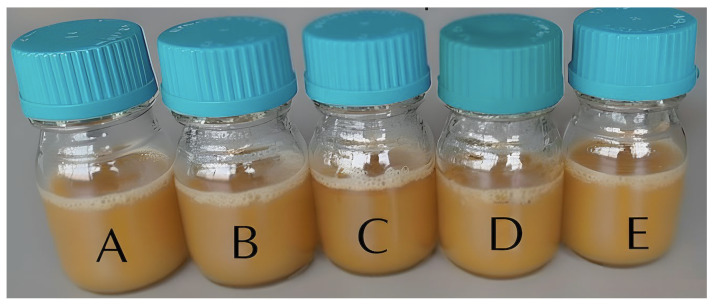
Unpasteurized apple juice without and with PPE microparticles systems: A: (J) juice; B: (J+PPE) juice with non-encapsulated pomegranate peel extract; C: (J+PPE-IN) juice with pomegranate peel extract-inulin microparticles; D: (J+PPE-(IN+SA)) juice with pomegranate peel extract-inulin-sodium alginate microparticles; E: (J+PPE-(IN+CR)) juice with pomegranate peel extract-inulin-carrageenan microparticles.

The MIC was determined to establish the concentration of non-encapsulated and encapsulated PPE to be added to unpasteurized apple juice. For this, non-encapsulated PPE was added in a concentration range of 2–12 mg PPE/mL of apple juice. Significant differences were found with a lower count from a concentration of 9 mg PPE/mL of apple juice after 24 h of incubation for RAM and 5 days for molds and yeasts. This concentration was selected for microbiological analysis. Figure 2 illustrates the apple juice systems, both with and without PPE microparticles, used in the microbiological study.

#### 3.3.1. MAB

**Figure 3:**
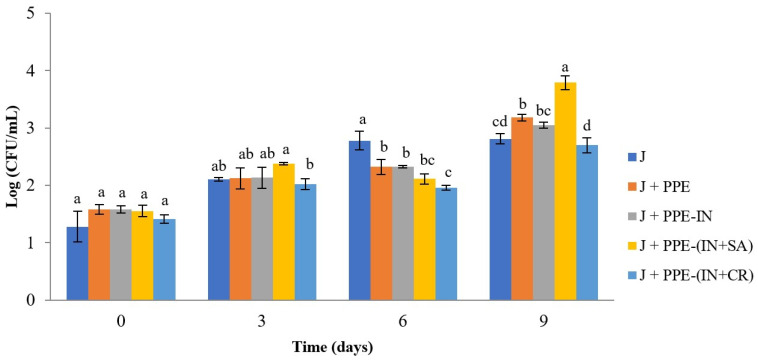
Mesophilic aerobic bacteria (MAB) counts in unpasteurized apple juice treated with non-encapsulated or encapsulated pomegranate peel extract (PPE) during refrigerated storage at 4 °C. CFU: colony-forming units; (J) juice; (J+PPE) juice with non-encapsulated pomegranate peel extract; (J+PPE-IN) juice with pomegranate peel extract-inulin microparticles; (J+PPE-(IN+SA)) juice with pomegranate peel extract-inulin-sodium alginate microparticles; (J+PPE-(IN+CR)) juice with pomegranate peel extract-inulin-carrageenan microparticles. Values are expressed as mean ± standard deviation (*n* = 3). Different lowercase letters indicate statistically significant differences between treatments at the same time point (*p* < 0.05).

The progression of MAB counts, expressed as Log (CFU/mL) over time, is shown in Figure 3. The initial MAB count in juice was 1.28 CFU/mL. As expected, all systems exhibited an increasing trend in MAB levels throughout the storage period.

There were no significant differences among the five systems at T_0_ (1.28 to 1.41 CFU/mL). Significant differences were only observed between J+PPE-(IN+SA) and J+PPE-(IN+CR) at T_3_. The latter presented the lowest MAB (2.02 CFU/mL), which was significantly lower than that of the other systems. The juice presented a significantly higher (2.78 CFU/mL) MAB count than J+PPE, J+PPE-IN, J+PPE-(IN+SA), and J+PPE-(IN+CR) systems; the latter showed the lowest (1.96 CFU/mL) at T_6_. Unlike T_9_, all systems increased their MAB count, with the highest value presented by the J+PPE-(IN+SA) system (3.79 CFU/mL) and the lowest by the J+PPE-(IN+CR) system (2.70 CFU/mL). Therefore, the lowest MAB in T_3_, T_6_, and T_9_ corresponded to J+PPE-(IN+CR) system.

The MAB count was below the values established by the Chilean regulation [51] for unpasteurized fruit juices (5 CFU/mL) during the measurement period (Figure 3). Note that either PPE (without encapsulation) or PPE microparticles showed higher AMA (i.e., lower MAB count) at T_6_ in relation to the juice, suggesting that PPE and PPE microparticles offer greater microbiological stability in juice systems. This AMA could be attributed to PPE itself in the J+PPE system and surface punicalagin in J+PPE-IN, J+PPE-(IN+SA), and J+PPE-(IN+CR) systems. Furthermore, among PPE microparticles, PPE-(IN+CR) showed differences with PPE at T_6_ and T_9_.

To our knowledge, there are no studies evaluating the antimicrobial activity of encapsulated PPE in juices. Furthermore, only one study shows the antimicrobial activity of PPE non-encapsulated. Selahvarzi et al. [20] investigated the effect of the orange peel extract and PPE as natural preservatives on functional drinks. Antimicrobial activity was tested in vitro using *S. aureus* and *E. coli*, and subsequently in a functional drink. The results showed that both extracts exhibited antimicrobial activity against bacteria; however, the orange peel extract demonstrated higher antimicrobial activity, reducing the growth of total bacteria, molds, and yeast. Besides, orange peel extract has no effect on the sensory properties of the drink compared to PPE. Rifna et al. [43] encapsulated pomegranate peel extract (PPE) using ionic gelation using sodium alginate. The antimicrobial activity of particles against *S. aureus* (ATCC 25923) in powdered infant formula was assessed. The results also showed that the antibacterial activity of the encapsulated PPE extracts against the target organism was comparable to that of commonly used synthetic antibiotics in food preservation.

Other studies have evaluated the antimicrobial activity of different bioactive compounds encapsulated against pathogenic bacteria. Rosemary essential oil was encapsulated with β-cyclodextrins to evaluate its antimicrobial effect against *S. typhimurium*, *L. monocytogenes*, *Candida tropicalis*, and *Saccharomyces pastorianus* in pasteurized tomato juice. The encapsulation of rosemary oil with β-cyclodextrins provided protection to its volatile constituents under high-temperature conditions and preserved its antimicrobial properties after both the encapsulation process and tomato juice pasteurization, compared to non-encapsulated rosemary oil [52]. Khan et al. [53] evaluated the antimicrobial effect of nisin nanoparticles against pathogenic microorganisms in orange juice, using chitosan and chemically modified chitosan as encapsulating agents. The results indicated that the efficacy of nisin was enhanced when encapsulated into nanoparticles, with a greater antimicrobial reduction observed for modified chitosan nanoparticles against both Gram-positive and Gram-negative bacterial strains.

#### 3.3.2. Molds and Yeasts

**Figure 4:**
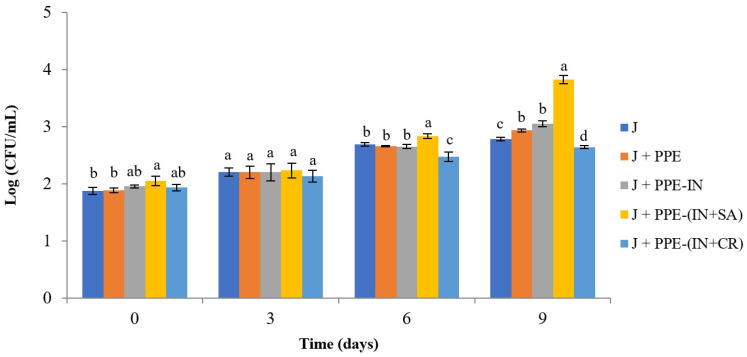
Mold and yeast counts in unpasteurized apple juice treated with non-encapsulated or encapsulated pomegranate peel extract (PPE) during refrigerated storage at 4 °C. CFU: colony-forming units; (J) juice; (J+PPE) juice with non-encapsulated pomegranate peel extract; (J+PPE-IN) juice with pomegranate peel extract-inulin microparticles; (J+PPE-(IN+SA)) juice with pomegranate peel extract-inulin-sodium alginate microparticles; (J+PPE-(IN+CR)) juice with pomegranate peel extract-inulin-carrageenan microparticles. Values are expressed as mean ± standard deviation (*n* = 3). Different lowercase letters indicate statistically significant differences between treatments at the same time point (*p* < 0.05).

The progression of molds and yeast counts, expressed as Log (CFU/mL) over time, is shown in Figure 4. The initial molds and yeast count in fresh apple juice was 1.87 (CFU/mL). Similar to the MAB trend, an increase in mold and yeast counts was observed in all systems over the storage period. At T_0_, the J+PPE-(IN+SA) system showed higher (2.05 CFU/mL) counts than the J+PPE (1.88 CFU/mL) treatment. No significant differences were found among the five systems at the T3 time point. At T_6_, J+PPE-(IN+SA) exhibited the highest (2.83 CFU/mL) mold and yeast counts, while J+PPE-(IN+CR) presented the lowest (2.47 CFU/mL). A similar pattern was observed at T_9_, with J+PPE-(IN+SA) showing the highest value (3.82 CFU/mL) and J+PPE-(IN+CR) showing the lowest value (2.64 CFU/mL).

Our results showed that mold and yeast counts followed a similar behavior to MAB counts. Therefore, it can be established that PPE-(IN+CR) microparticles provide greater microbiological stability of juice up to T_9_, presenting significantly lower MAB and molds and yeast count compared to the other systems within the established regulatory limits.

When microencapsulated bioactive compounds exhibit rapid release kinetics, the active compound becomes prematurely exposed to the food matrix, losing its protective effect and, consequently, reducing its efficacy. This type of release is known as a burst effect. Robert et al. [32] reported that the release of gallic acid in water at pH 5.0 exhibited this behavior, indicating that the compound was released from inulin microparticles through a non-Fickian diffusion mechanism. A similar behavior could have occurred in the J+(PPE-IN) system, as the microbiological activity observed was comparable to that of juice with non-encapsulated PPE on days 6 and 9 of storage.

For this reason, in the present study, a mixture of IN with either SA or CR was also used in order to create a diffusion barrier that slows the release of active compounds, depending on the characteristics of each polymer. In this context, different pH levels can alter the degree of dissociation and/or the conformation of the encapsulating agent, allowing for greater or lesser control over the release of the encapsulated bioactive compounds [32]. In the J+PPE-(IN+SA) system, SA is a polymer that remains stable at acidic pH and forms a gel structure, whereas under alkaline conditions it swells and rapidly disintegrates, releasing the encapsulated bioactive compounds [54]. For this reason, SA has been widely used in microencapsulation technology for the controlled release of bioactive compounds. Thus, in the J+(PPE-SA) system, the PPE may not be released, or may be released very slowly, as reflected by the highest counts observed on day 9.

In the J+PPE-(IN+CR) system, CR may become gradually destabilized under acidic pH conditions, leading to a progressive opening of its polymeric structure, which allows for the controlled diffusion of PPE into the acidic apple juice matrix. Consequently, PPE release would not occur as a single initial “burst,” as observed for non-encapsulated PPE and the J+PPE-IN system, but rather as a prolonged and/or controlled release. Additionally, microencapsulation reduces the direct interaction between the extract and juice components, which could otherwise inactivate phenolic compounds. Thus, PPE could exert a sustained antimicrobial effect throughout the 9-day storage period, significantly extending the product’s shelf life compared with the other systems evaluated.

It is important to note that the release of a bioactive compound from microparticles depends on the nature of both the encapsulating agent and the active compound, as well as on the surrounding medium or food matrix, which collectively determine the release rate and mechanism [32]. In the present study, the type of biopolymer used as the encapsulating agent, either individually or in combination, had a significant effect on the antimicrobial activity of the encapsulated polyphenols in apple juice.

## 4. Conclusions

The microbiological stability of unpasteurized fresh apple juice, adding non-encapsulated PPE or PPE microparticles, showed differences according to the type of PPE microparticles. Our hypothesis was partially confirmed, as the MAB, mold, and yeast counts were significantly lower in the J+ PPE-(IN+CR) system than in the J+PPE and microparticle systems at T_6_ and T_9_. Therefore, adding PPE-(IN+CR) microparticles into unpasteurized fresh apple juice provided greater microbiological stability during the study. These results reinforce the idea that recycling pomegranate by-products can be used to design natural food additives with antimicrobial properties, and furthermore, provide a solution to reduce waste generation along food systems. This aligns with Sustainable Development Goal (SDG) 12.5: “By 2030, substantially reduce waste generation through prevention, reduction, recycling, and reuse” (United Nations, 2015) [55]. Future studies should focus on analyzing impacts on the sensory, physicochemical, or nutritional properties of the juice, such as flavor, color, or vitamin content, and understanding the kinetics and mechanism of release of PPE-IN, PPE-(IN+SA), or PPE-(IN+CR) microparticles to define their most favorable applications in foods.

## Figures and Tables

**Table 1:** Experimental conditions and results from an orthogonal central composite design for the microencapsulation by spray-drying of pomegranate peel extract (PPE) with inulin.

Run	PPE/IN Ratio	Inlet Air Temperature (°C)	EE (%)
1	1:0.5	120	83.3 ± 2.3
2	1:0.5	190	93.1 ± 0.8
3	1:2	120	90.7 ± 0.8
4	1:2	190	95.2 ± 0.9
5	1:1.25	112.65	89.0 ± 0.5
6	1:1.25	197.35	94.3 ± 0.2
7	1:0.34	155	86.8 ± 3.4
8	1:2.16	155	93.1 ± 0.2
9	1:1.25	155	91.5 ± 1.1
10	1:1.25	155	91.7 ± 1.4
11	1:1.25	155	92.3 ± 1.4
12	1:1.25	155	92.8 ± 1.7

PPE = pomegranate peel extract; IN = inulin; EE = encapsulation efficiency. Values are expressed as mean ± standard deviation (*n* = 3). R-squared (adjusted for d.f.) = 94.0229 percent. Lack of fit= 2.49. Residual value < 1.7.

**Table 2:** Characterization of optimal microparticles containing pomegranate peel extract (PPE) obtained by spray-drying with different encapsulating agents.

Microparticle System	PPE-IN	PPE-(IN+SA)	PPE-(IN+CR)
PPE:EA ratio	1:2.1	1:2.1	1:2.1
Inlet air temperature (ºC)	166	166	166
Total solids (g)	15.5	15.5	15.5
Water activity	0.18 ± 0.01 ^a^	0.15 ± 0.01 ^ab^	0.14 ± 0.02 ^b^
Moisture content (%)	3.46 ± 0.21 ^a^	2.72 ± 0.16 ^b^	3.63 ± 0.21 ^a^
Total punicalagin content (mg/g)	4.45 ± 0.23 ^a^	3.59 ± 0.33 ^b^	3.853 ± 0.004 ^b^
Surface punicalagin content (mg/g)	0.26 ± 0.01 ^b^	0.35 ± 0.03 ^a^	0.32 ± 0.02 ^a^
Encapsulation efficiency (%)	94.08 ± 0.58 ^a^	90.06 ± 1.74 ^b^	91.59 ± 0.48 ^ab^
Mean particle diameter ((D4.3) µm)	5.87	11.69	13.30
D90 (µm)	10.19	22.46	26.92

PPE: pomegranate peel extract; EA: encapsulating agent; IN: inulin; SA: sodium alginate; CR: carrageenan. PPE-IN: pomegranate peel extract inulin microparticles; PPE-(IN+SA): pomegranate peel extract inulin + sodium alginate microparticles; PPE-(IN+CR): pomegranate peel extract inulin + carrageenan microparticles. Values are expressed as mean ± standard deviation (*n* = 3). Different lowercase letters indicate statistically significant differences (*p* < 0.05) between microparticle systems.

## Data Availability

The original contributions presented in this study are included in the article. Further inquiries can be directed to the corresponding author.
